# Cerebrospinal fluid metallomics in cerebral amyloid angiopathy: an exploratory analysis

**DOI:** 10.1007/s00415-021-10711-6

**Published:** 2021-07-22

**Authors:** Gargi Banerjee, Niklas Forsgard, Gareth Ambler, Ashvini Keshavan, Ross W. Paterson, Martha S. Foiani, Jamie Toombs, Amanda Heslegrave, Edward J. Thompson, Michael P. Lunn, Nick C. Fox, Henrik Zetterberg, Jonathan M. Schott, David J. Werring

**Affiliations:** 1 Department of Brain Repair and Rehabilitation, Stroke Research Centre, UCL Queen Square Institute of Neurology, Russell Square House, 10-12 Russell Square, London, WC1B 5EH UK; 2 National Hospital for Neurology and Neurosurgery, Queen Square, London, UK; 3 Department of Clinical Chemistry, Sahlgrenska University Hospital, Gothenburg, Sweden; 4 Department of Statistical Science, University College London, London, UK; 5 Department of Neurodegenerative Disease, Dementia Research Centre, UCL Queen Square Institute of Neurology, London, UK; 6 Department of Neurodegenerative Disease, UCL Queen Square Institute of Neurology, London, UK; 7 UK Dementia Research Institute at UCL, London, UK; 8 Neuroimmunology and CSF Laboratory (NICL), UCL Queen Square Institute of Neurology, London, UK; 9 MRC Centre for Neuromuscular Disease, National Hospital for Neurology and Neurosurgery, London, UK; 10 Clinical Neurochemistry Laboratory, Sahlgrenska University Hospital, Mölndal, Sweden; 11 Department of Psychiatry and Neurochemistry, Institute of Neuroscience and Physiology, The Sahlgrenska Academy at the University of Gothenburg, Mölndal, Sweden

**Keywords:** Alzheimer’s disease, Cerebral amyloid angiopathy, Cerebrospinal fluid, Ferritin, Metallomics, Iron

## Abstract

**Introduction:**

Cerebral amyloid angiopathy (CAA) is associated with symptomatic intracerebral haemorrhage. Biomarkers of clinically silent bleeding events, such as cerebrospinal fluid (CSF) ferritin and iron, might provide novel measures of disease presence and severity.

**Methods:**

We performed an exploratory study comparing CSF iron, ferritin, and other metal levels in patients with CAA, control subjects (CS) and patients with Alzheimer’s disease (AD). Ferritin was measured using a latex fixation test; metal analyses were performed using inductively coupled plasma mass spectrometry.

**Results:**

CAA patients (*n* = 10) had higher levels of CSF iron than the AD (*n* = 20) and CS (*n* = 10) groups (medians 23.42, 15.48 and 17.71 μg/L, respectively, *p* = 0.0015); the difference between CAA and AD groups was significant in unadjusted and age-adjusted analyses. We observed a difference in CSF ferritin (medians 10.10, 7.77 and 8.01 ng/ml, for CAA, AD and CS groups, respectively, *p* = 0.01); the difference between the CAA and AD groups was significant in unadjusted, but not age-adjusted, analyses. We also observed differences between the CAA and AD groups in CSF nickel and cobalt (unadjusted analyses).

**Conclusions:**

In this exploratory study, we provide preliminary evidence for a distinct CSF metallomic profile in patients with CAA. Replication and validation of these results in larger cohorts is needed.

**Supplementary Information:**

The online version contains supplementary material available at 10.1007/s00415-021-10711-6.

## Introduction

Cerebral amyloid angiopathy (CAA) is a cerebral small vessel disease characterised by amyloid-beta (Aβ) deposition in cortical and leptomeningeal blood vessels [4]. CAA is associated with lobar intracerebral haemorrhage; this, together with other haemorrhagic imaging markers (cerebral microbleeds, cortical superficial siderosis), forms the basis for in vivo diagnosis [8, 11, 14]. Cerebrospinal fluid (CSF) biomarkers of clinically silent bleeding events might provide a novel means of quantifying disease presence and severity; however, data in CAA are limited [13].

We performed a pilot study investigating CSF iron and ferritin levels in patients with CAA, control subjects (CS) and patients with Alzheimer’s disease (AD), and how these correlated with other neurodegenerative CSF biomarkers (Aβ-40, Aβ-42, total tau, phospho-tau, and neurofilament light). We hypothesised that CSF ferritin and iron would be highest in the CAA group. In exploratory analyses, we measured concentrations of other CSF metals (nickel, chromium, zinc, manganese, cobalt and copper).

## Methods

### Patient selection

We included samples from the cross-sectional prospective observational BOCAA (Biomarkers and Outcomes in Cerebral Amyloid Angiopathy) study [5], and samples collected by the Specialist Cognitive Disorders Service at the National Hospital for Neurology and Neurosurgery, London, UK; group inclusion and exclusion criteria and the standardised protocol for sample collection have been provided in detail previously [3] and therefore will only be described briefly here. Patients with CAA all met “probable” modified Boston Criteria [11] and had been asymptomatic (i.e. without clinical evidence of acute intracerebral haemorrhage) in the 6 months prior to their study visits; CSF biomarkers were not used in the diagnostic process. Patients with AD had an amnestic presentation and were diagnosed on the basis of clinical assessment, imaging, and CSF measures; those with haemorrhagic imaging markers of CAA (cerebral microbleeds, cortical superficial siderosis) were excluded. Control subjects had no significant neurological diagnoses and had no evidence of significant cerebrovascular disease (including CAA) or atrophy on brain (MR) head imaging. Informed written consent was obtained for all participants.

### CSF analysis

Methods for CSF processing, including measurement of Aβ-40, Aβ-42, total tau, phospho-tau and neurofilament light, have been described previously [3].

Ferritin was measured using  a latex fixation test according to manufacturer’s instructions (described previously) [9]. All samples were measured on the same day by a single operator using the same reagents.

CSF metal analyses were performed by inductively coupled plasma mass spectrometry (ICP-MS) with an octopole reaction system operated in the helium collision mode (Agilent 7700 × ICP-MS; Agilent Technologies, Santa Clara, Ca, USA). All samples were diluted 10 times with a basic diluent containing 1-butanol (2%w/v), ethylenediaminetetraacetic acid (EDTA) (0.05%w/v), Triton X-100 (0.05%w/v), and ammonium hydroxide (1%w/v) and were analysed in a single batch after calibration performed in the dilution medium. Germanium was used as internal standard for all elements. One quality control sample was analysed in the beginning and end of the batch (Seronorm™ Trace Elements Urine L-1, Lot no. 1011644); all element concentrations were within the stated acceptable limits.

### Statistics

Statistical analysis was performed using Stata (Version 15.1). Group characteristics were compared using either one-way ANOVA (normally distributed variables), Chi-squared (categorical variables) or Kruskal–Wallis (non-normally distributed data, including all biomarkers) tests. For biomarkers, if a significant difference was identified (defined as *p* < 0.05), Dunn’s test was used for post hoc comparisons, and a Bonferroni correction (resultant *p* value multiplied by 3) applied.

In order to perform age-adjusted analyses, we used quantile regression (comparing group medians) and calculated predicted medians. We then performed post hoc pairwise comparisons of the age-adjusted medians; statistical significance was defined as Bonferroni-corrected *p* < 0.05.

Spearman correlation was used to look for correlations between CSF ferritin and iron and other neurodegenerative CSF markers. Associations with a Spearman’s rho value (*ρ*) above 0.3 or below − 0.3 were regarded as correlations of potential interest.

## Results

We included 10 patients with CAA, 20 patients with AD and 10 control subjects (Table 1). Patients with CAA were older than the two other groups, and patients with AD had lower mini-mental state examination (MMSE) scores.

**Table 1:** Comparison of characteristics and biomarkers by group

	CAA (*n* = 10)	AD (*n* = 20)	CS (*n* = 10)	Group comparison, *p* value	Post hoc comparisons; *p* values		
					CAA/AD	CAA/CS	AD/CS
Age, years, mean (SD)	68.6 (3.0)	62.5 (4.1)	62.2 (5.4)	0.001	–	–	–
Sex, female, *n* (%)	2 (20%)	11 (55%)	5 (50%)	0.18	–	–	–
MMSE, median (IQR)	29 (28 to 30)	24 (19.5 to 26)	29 (29 to 30)	< 0.001	–	–	–
*CSF biomarkers*							
Ferritin, ng/ml, median (IQR)	10.10 (8.37 to 14.00)	7.77 (6.39 to 9.18)	8.01 (6.94 to 8.95)	0.0136	0.0060	0.0483	1.0000
Iron, concentration (μg/L), median (IQR)	23.42 (18.82 to 25.30)	15.48 (12.28 to 18.26)	17.71 (15.19 to 20.76)	0.0015	0.0006	0.0912	0.2268

Group comparison *p* values were obtained using one-way ANOVA (age), Chi-squared tests (sex), or Kruskal–Wallis tests (remainder). Post hoc comparisons were made using Dunn’s test; the presented *p* values are Bonferroni-corrected

*AD* Alzheimer’s disease, *CAA* cerebral amyloid angiopathy, *CS* control subjects, *IQR* interquartile range, *MMSE* mini-mental state examination, *SD* standard deviation

**Fig. 1:**
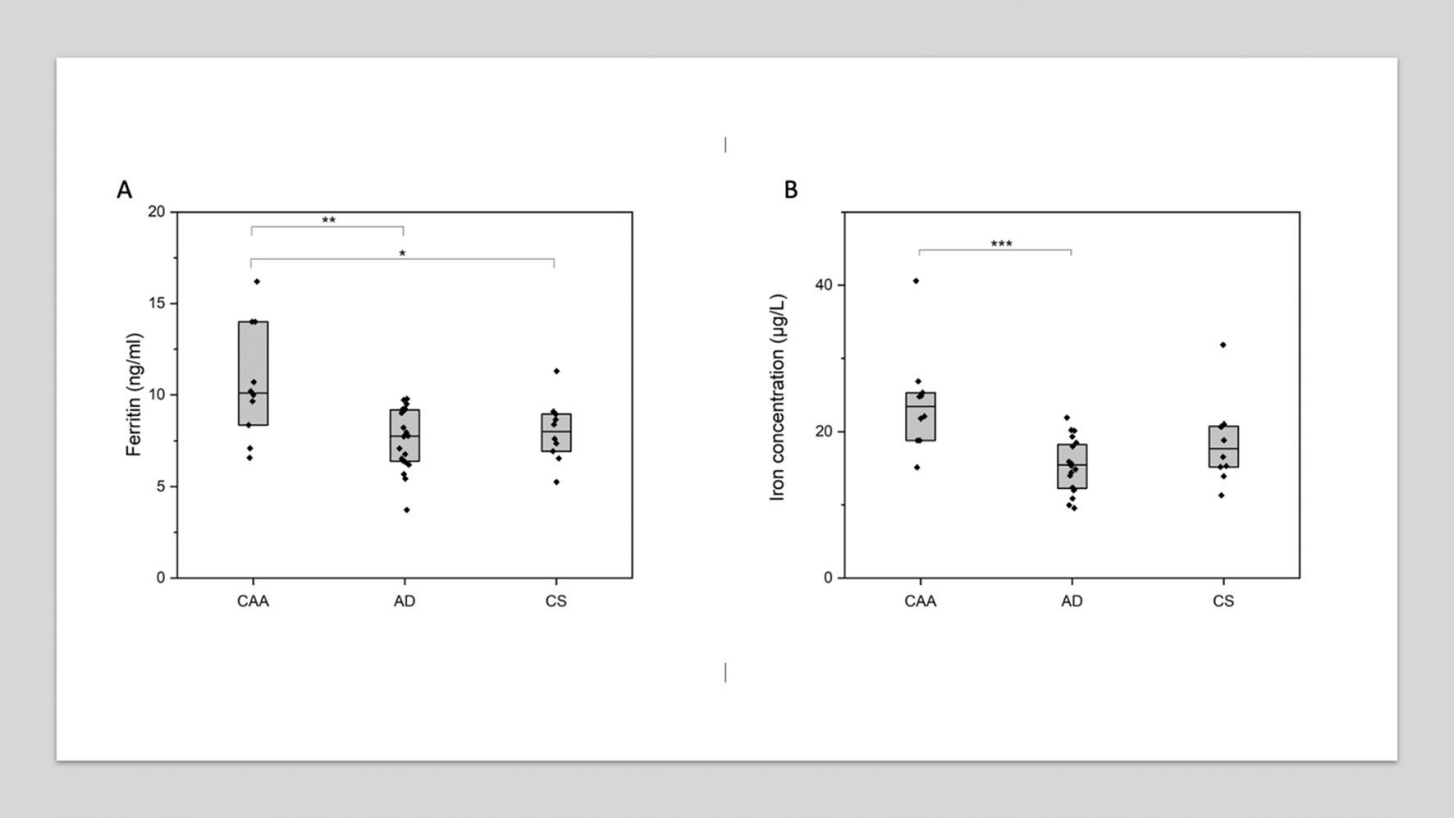
CSF ferritin (**A**) and iron (**B**) by group. Horizontal line indicates median value per group; box shows 25th and 75th percentile. Each diamond indicates an individual data point. *p* values are derived from post hoc Dunn’s test and have been Bonferroni-corrected. *Indicates *p* ≤ 0.05; **indicates *p* ≤ 0.01; ***indicates *p* ≤ 0.001. *AD* Alzheimer’s disease, *CAA* cerebral amyloid angiopathy, *CS* control subjects

In univariable comparisons (Table 1, Fig. 1), patients with CAA had higher CSF ferritin levels than the other groups (median 10.10 ng/ml in CAA group vs 7.77 ng/ml in AD group and 8.01 ng/ml in the CS group, *p* = 0.01). Patients with CAA also had significantly higher CSF iron levels than the AD group (median 23.42 μg/L, vs 15.48 μg/L, *p* = 0.0006); the difference in medians between the CAA and CS group was of a similar magnitude (median 23.42 µg/L vs 17.71 µg/L), but did not reach statistical significance (*p* = 0.09). There were no significant differences in CSF ferritin or iron when comparing the AD and CS groups.

In age-adjusted quantile regression (Supplementary Table 1), there was a significant difference in CSF iron between the CAA and AD groups (median difference − 7.11 μg/L), but no significant difference in CSF ferritin.

We then looked for correlations between CSF ferritin and iron and other CSF biomarkers observed in neurodegenerative disease (Supplementary Table 2, Supplementary Fig. 1). When considering all groups together, there was a significant correlation between CSF Aβ-42 and CSF ferritin (Spearman’s *ρ* − 0.5057, *p* = 0.0009); there was a modest correlation between CSF ferritin and Aβ-40 (Spearman’s *ρ* − 0.3068, *p* = 0.054), although this did not quite reach statistical significance by our definition. There was evidence of a modest correlation between CSF Aβ-42 and CSF iron (Spearman’s ρ − 0.3122, *p* = 0.0499).

### Other CSF metals

We performed further exploratory analyses for other CSF metals, the results of which are provided in the Online Supplementary Material (Supplementary Tables 3 and 4; Supplementary Fig. 2). Briefly, there were differences between the CAA and AD groups in CSF nickel (median 0.39 μg/L vs 0.16 μg/L, *p* = 0.03), cobalt (median 0.03 μg/L vs 0.01 μg/L, *p* = 0.003); and manganese (median 1.19 μg/L vs 0.84 μg/L), although the latter was not quite of statistical significance (*p* = 0.05). In age-adjusted analyses, only the difference in CSF nickel remained of statistical significance (median difference − 0.25 μg/L).

## Discussion

In this pilot study, we provide evidence for a distinct CSF metallomic profile in patients with CAA, with higher levels of CSF iron observed in unadjusted and age-adjusted analyses. We also observed higher levels of CSF ferritin in the CAA group, reaching significance in unadjusted but not age-adjusted analyses. When considering correlations with CSF biomarkers associated with neurodegenerative disease, negative correlations of interest were observed with Aβ-40 (CSF ferritin), and Aβ-42 (CSF ferritin, CSF iron); together these measures might reflect the degree of underlying Aβ pathology (Supplementary Fig. 1), and a combination might provide useful diagnostic information in differentiating AD and CAA. In further exploratory analyses, we found differences of potential interest between the AD and CAA groups in CSF nickel, cobalt and manganese. CSF metallomic profile might therefore provide a novel means to differentiate between disease secondary to parenchymal versus vascular Aβ.

We hypothesise that the elevation in CSF iron observed in CAA is due to small asymptomatic bleeding events into the subarachnoid space, which later evolve on MRI into haemorrhagic features such as cortical superficial siderosis and superficially located lobar cerebral microbleeds. In aneurysmal subarachnoid haemorrhage, iron is released into the CSF following the haemolysis of extravasated erythrocytes and subsequent degradation of haemoglobin [12]. In this context, iron is toxic, and ferritin is produced intrathecally within 24 to 48 h of a haemorrhage in order to sequester the free iron [9, 12]. CSF ferritin levels in aneurysmal subarachnoid haemorrhage peak after 7 to 10 days, and can remain elevated for up to 2 months [9]. Cortical superficial siderosis in CAA develops after episodes of acute convexity subarachnoid bleeding [4], but provides no temporal information about when these haemorrhages occurred. A recent study [15] in a memory clinic population did not find any association between CSF iron and haemorrhagic MRI markers of CAA; this might reflect a lower frequency of CAA-related bleeding events (asymptomatic or symptomatic) in a memory clinic cohort than that expected in a stroke cohort, where haemorrhagic features might be more apparent. Although our observed difference in CSF ferritin was not significant in age-adjusted analyses, this might reflect a lack of power due to our small group sizes, given the biologically plausible mechanism for this observation. CSF iron and ferritin might be attractive new dynamic biomarkers for recent bleeding and thus haemorrhagic disease activity in an individual with CAA; future work is needed to explore this further.

Data from patients with AD have suggested that iron might have a role in accelerating disease progression. CSF ferritin has been associated with brain hypometabolism [7], and more rapid deteriorations in cognitive performance [2] and CSF Aβ-42 levels [1, 15]. Moreover, iron is thought to enhance Aβ production and pathogenicity via a number of mechanisms [10]. In our work, as in these previous reports, we observed that CSF ferritin and Aβ-42 were (negatively) correlated. However, we did not detect differences between controls and AD patients in CSF ferritin or iron; of the three groups in our study, levels of ferritin and iron were lowest in the AD group, although this was only statistically significant in comparisons (unadjusted for ferritin, unadjusted and age-adjusted for iron) with the CAA group. Our data might therefore suggest that previously described associations between CSF ferritin and poor outcomes in AD reflect the coexistence (and severity) of CAA [1, 7]. This hypothesis is supported by neuropathological data demonstrating that CAA has an independent negative effect on cognition in AD patients, independently of AD pathology [6]. Future studies should quantify the degree of CAA in AD patients and the impact this has on CSF iron and ferritin.

In our exploratory analyses for other CSF metals, we identified potential differences of interest between the CAA and AD groups in CSF nickel, manganese, and cobalt. Although there are some data for these and other CSF metals in AD, to our knowledge similar findings have not been described in CAA. This might suggest that CAA results in a more widespread disruption in metal homeostasis, but the clinical and mechanistic relevance of these results are unclear; again, replication and further study in larger cohorts is needed.

We acknowledge that our exploratory study is small and therefore potentially underpowered; however, our aim was to provide hypothesis-generating data for novel biomarkers of CAA. Our preliminary data suggest that CAA might result in a distinct CSF metallomic profile; replication and validation of these results in larger cohorts is needed.

## Supplementary Information

Below is the link to the electronic supplementary material.

Supplementary file1 (TIFF 3421 KB)

Supplementary file2 (TIFF 3421 KB)

Supplementary file3 (DOCX 28 KB)

## Data Availability

Additional data relating to the analyses presented in this manuscript are provided in the Supplementary Material. Analyses for the BOCAA study are ongoing; once all of these analyses are completed, the Chief Investigator (DJW) will consider applications from other researchers for access to anonymised source data.
