# Supplementary figures and images for: Cerebrospinal fluid metallomics in cerebral amyloid angiopathy: an exploratory analysis
Source: J Neurol. 2021 Jul 22;269(3):1470–5. doi: 10.1007/s00415-021-10711-6 (PMC8857160; doi:10.1007/s00415-021-10711-6)

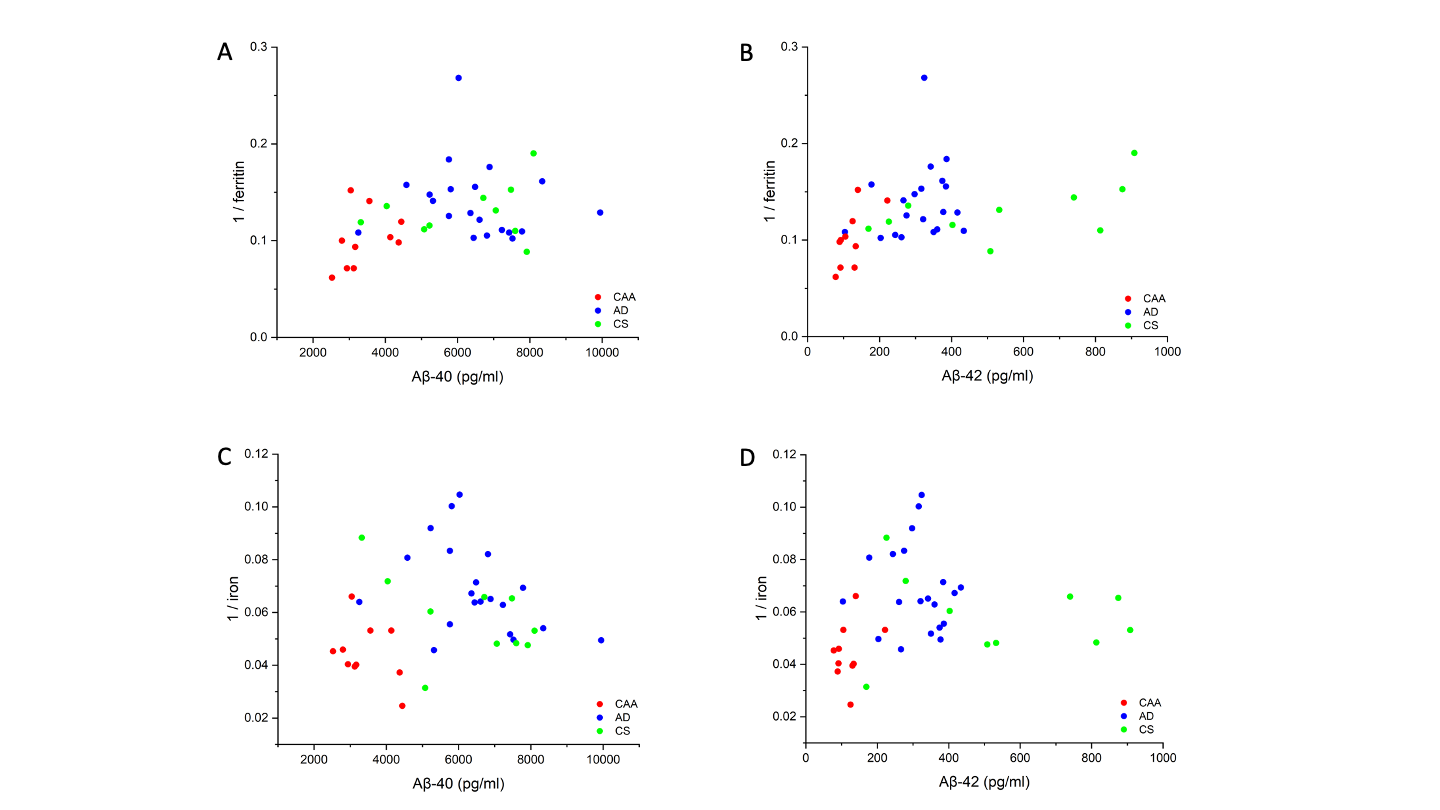

Supplement: Supplementary file 1 — Supplementary file1 (TIFF 3421 KB) [file 415_2021_10711_MOESM1_ESM.tiff]

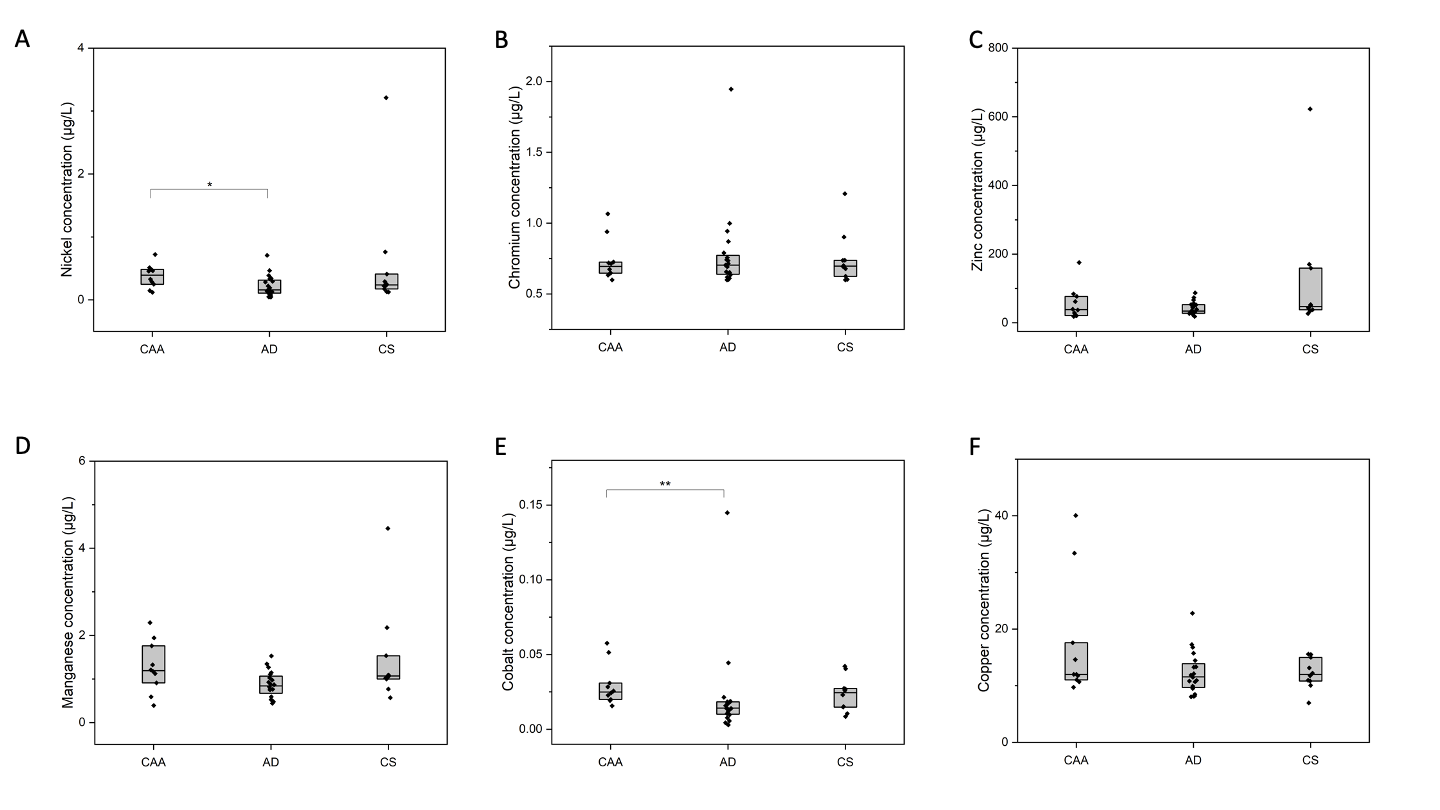

Supplement: Supplementary file 2 — Supplementary file2 (TIFF 3421 KB) [file 415_2021_10711_MOESM2_ESM.tiff]
