# Supplementary material for: Cerebrospinal fluid metallomics in cerebral amyloid angiopathy: an exploratory analysis
Source: J Neurol. 2021 Jul 22;269(3):1470–5. doi: 10.1007/s00415-021-10711-6 (PMC8857160; doi:10.1007/s00415-021-10711-6)
Supplement: Supplementary file 3 — Supplementary file3 (DOCX 28 KB) [file 415_2021_10711_MOESM3_ESM.docx]

**ONLINE SUPPLEMENTARY MATERIAL**

**SUPPLEMENTARY TABLES**

Supplementary Table 1: Age-adjusted quantile regression (comparing medians) for CSF ferritin and iron

* p<0.05

Abbreviations: AD, Alzheimer’s disease; CAA, cerebral amyloid angiopathy; CS, control subjects; IQR, interquartile range; SE, standard error.

| **Biomarker** | **Group** | **Age-adjusted difference in medians (SE)** | **p value** |
| --- | --- | --- | --- |
| Ferritin, ng/ml, median (IQR) | CAA | *Reference group* | 0.4654 |
|  | AD | -1.67 (1.39) |  |
|  | CS | -0.90 (1.56) |  |
| Iron, concentration (μg/L), median (IQR) | CAA | *Reference group* | 0.0068 |
|  | AD | -7.11 (2.14) * |  |
|  | CS | -4.19 (2.42) |  |

**Supplementary** **Table 2: Correlations between CSF ferritin and iron) and other neurodegenerative CSF biomarkers**

Comparisons made using Spearman correlation.

Abbreviations: Aβ, amyloid beta; AD, Alzheimer’s disease; CAA, cerebral amyloid angiopathy; CS, control subjects; ρ, Spearman’s rho.

|  | **Whole cohort (AD / CAA / CS; n=40)** | | | | **CAA only (n=10)** | | | |
| --- | --- | --- | --- | --- | --- | --- | --- | --- |
|  | **Ferritin** | | **Iron** | | **Ferritin** | | **Iron** | |
| **Biomarker** | **ρ** | **p value** | **ρ** | **p value** | **ρ** | **p value** | **ρ** | **p value** |
| Aβ-40 | -0.3068 | 0.0542 | -0.1576 | 0.3314 | -0.4681 | 0.1725 | 0.3939 | 0.2600 |
| Aβ-42 | -0.5057 | 0.0009 | -0.3122 | 0.0499 | -0.6383 | 0.0470 | -0.2970 | 0.4047 |
| Total tau | -0.0243 | 0.8817 | -0.0081 | 0.9606 | 0.3708 | 0.2915 | 0.5636 | 0.0897 |
| Phospho-tau | 0.0122 | 0.9405 | 0.0612 | 0.7077 | 0.2492 | 0.4874 | 0.5515 | 0.0984 |
| Neurofilament light | 0.2394 | 0.1368 | 0.2887 | 0.0708 | -0.0547 | 0.8807 | 0.0545 | 0.8810 |

Supplementary Table 3: Comparison of other CSF metals

Group comparison p values were obtained using Kruskal-Wallis tests. Post-hoc comparisons were made using Dunn’s test; the presented p values are Bonferroni corrected.

Abbreviations: AD, Alzheimer’s disease; CAA, cerebral amyloid angiopathy; CS, control subjects; IQR, interquartile range; MMSE, mini-mental state examination; SD, standard deviation.

|  | **CAA (n=10)** | **AD (n=20)** | **CS (n=10)** | **Group comparison, p value** | **Post-hoc comparisons;**  **p values** | | |
| --- | --- | --- | --- | --- | --- | --- | --- |
|  |  |  |  |  | **CAA / AD** | **CAA / CS** | **AD / CS** |
| Nickel, concentration (μg/L), median (IQR) | 0.39 (0.25 to 0.48) | 0.16 (0.11 to 0.32) | 0.24 (0.17 to 0.41) | 0.0488 | 0.0297 | 0.7188 | 0.1956 |
| Chromium, concentration (μg/L), median (IQR) | 0.69 (0.65 to 0.73) | 0.70 (0.64 to 0.77) | 0.70 (0.62 to 0.74) | 0.9706 | - | - | - |
| Zinc, concentration (μg/L), median (IQR) | 38.31 (21.18 to 76.74) | 34.28 (27.49 to 52.72) | 47.28 (38.10 to 159.12) | 0.2598 | - | - | - |
| Manganese, concentration (μg/L), median (IQR) | 1.19 (0.91 to 1.76) | 0.84 (0.67 to 1.07) | 1.07 (1.00 to 1.53) | 0.0539 | - | - | - |
| Cobalt, concentration (μg/L), median (IQR) | 0.03 (0.02 to 0.03) | 0.01 (0.01 to 0.02) | 0.02 (0.01 to 0.03) | 0.0052 | 0.0027 | 0.4134 | 0.0930 |
| Copper, concentration (μg/L), median (IQR) | 12.00 (11.03 to 17.58) | 11.56 (9.71 to 13.88) | 11.98 (10.82 to 14.99) | 0.4456 | - | - | - |

Supplementary Table 4: Age-adjusted quantile regression (comparing medians) for other CSF metals

* p<0.05

Abbreviations: AD, Alzheimer’s disease; CAA, cerebral amyloid angiopathy; CS, control subjects; IQR, interquartile range; SE, standard error.

| **Biomarker** | **Group** | **Age-adjusted difference in medians (SE)** | **p value** |
| --- | --- | --- | --- |
| Nickel, concentration (μg/L), median (IQR) | CAA | *Reference group* | 0.0372 |
|  | AD | -0.25 (0.09)* |  |
|  | CS | -0.17 (0.10) |  |
| Chromium, concentration (μg/L), median (IQR) | CAA | *Reference group* | 0.9290 |
|  | AD | -0.03 (0.08) |  |
|  | CS | -0.03 (0.09) |  |
| Zinc, concentration (μg/L), median (IQR) | CAA | *Reference group* | 0.8272 |
|  | AD | -0.69 (21.42) |  |
|  | CS | 10.34 (24.15) |  |
| Manganese, concentration (μg/L), median (IQR) | CAA | *Reference group* | 0.2372 |
|  | AD | -0.40 (0.26) |  |
|  | CS | -0.14 (0.29) |  |
| Cobalt, concentration (μg/L), median (IQR) | CAA | *Reference group* | 0.0935 |
|  | AD | -0.01 (0.01) |  |
|  | CS | 0.00 (0.01) |  |
| Copper, concentration (μg/L), median (IQR) | CAA | *Reference group* | 0.9974 |
|  | AD | -0.03 (1.87) |  |
|  | CS | 0.09 (2.11) |  |

**SUPPLEMENTARY FIGURES**

**Supplementary Figure 1: Scatter plots of CSF Aβ measures against reciprocal values for CSF ferritin and iron**

A, CSF Aβ-40 against 1 / CSF ferritin; B, CSF Aβ-42 against 1 / CSF ferritin; C, CSF Aβ-40 against 1 / CSF iron; D, CSF Aβ-42 against 1 / CSF iron.

**Supplementary Figure 2: Comparison of other CSF metals**

A, Nickel; B, Chromium; C, Zinc; D, Manganese; E, Cobalt; F, Copper.

Horizontal line indicates median value per group; box shows 25th and 75th percentile.

Each diamond indicates an individual data point.

p values are derived from post-hoc Dunn’s test and have been Bonferroni-corrected.

* indicates p ≤ 0.05

** indicates p ≤ 0.01

Abbreviations: AD, Alzheimer’s disease; CAA, cerebral amyloid angiopathy; CS, control subjects.
